# Examining emotional and behavioural trajectories in siblings of children with life-limiting conditions

**DOI:** 10.1186/s12904-024-01535-y

**Published:** 2024-08-12

**Authors:** Joanne Tay, Kimberley Widger, Rose Steele, Robyn Stremler, Jason D. Pole

**Affiliations:** 1 Faculty of Nursing, University of Windsor, Room 317, Toldo Health Education Centre, 401 Sunset Avenue, Windsor, ON N9B 3P4 Canada; 2 Lawrence S. Bloomberg Faculty of Nursing, University of Toronto, Toronto, Canada; 3 Paediatric Advanced Care Team, Hospital for Sick Children, Toronto, Canada; 4 Life Stage Program, ICES, Toronto, Canada; 5 School of Nursing, York University, Toronto, Canada; 6 Child Health Evaluative Sciences, Hospital for Sick Children, Toronto, Canada; 7 Centre for Health Services Research, The University of Queensland, Brisbane, Australia; 8 Cancer Research Program, ICES, Toronto, Canada; 9 Dalla Lana School of Public Health, University of Toronto, Toronto, Canada

**Keywords:** Child behaviour, Sibling(s), Children with medical complexity, Linear mixed effect model

## Abstract

**Background:**

Healthy siblings of children with life-limiting conditions often experience emotional and behavioural struggles over the course of the ill child’s condition(s). Resources to support these siblings are limited due to a lack of understanding about their needs. Therefore, this study was designed to characterize the emotional and behavioural trajectories among siblings of children with progressive, life-limiting genetic, metabolic, or neurological conditions over a 12-month observation period.

**Methods:**

Seventy siblings were recruited from a large-survey based study (Charting the Territory) that examined the bio-psychosocial health outcomes of parents and siblings. Linear mixed effect models were used to assess the association between siblings’ emotions and behaviour trajectories and selected demographic variables. Siblings’ emotions and behaviour were measured with Child Behaviour Checklist (CBCL).

**Results:**

Siblings’ mean age was 11.2 years at baseline and Internalizing, Externalizing, and Total Behaviour Problems mean scores were within normal ranges across time. However, 7–25% of siblings had scores within the clinical range. Brothers had higher levels of Internalizing Problems than sisters, whereas sisters had higher levels of Externalizing Problems than brothers. When treatment was first sought for the ill child less than a year prior to study participation, siblings had higher levels of Internalizing and Externalizing Problems compared with siblings who participated more than one year after treatment was sought.

**Conclusion:**

Healthy siblings experience emotional and behavioural problems early in the child’s disease trajectory. Although these problems improve with time, our findings show that brothers and sisters experience different types of challenges. Therefore, timely support for siblings is important as they navigate through the uncertainties and challenges.

**Supplementary Information:**

The online version contains supplementary material available at 10.1186/s12904-024-01535-y.

## Introduction

An estimated 21 million children worldwide are living with life-limiting conditions [1]. Together for Short Lives defined life-limiting conditions in children and youth according to four categories: (1) conditions for which curative treatment may be feasible but can fail, for example, cancer; (2) conditions where premature death is inevitable but require long periods of intensive treatment to partake in everyday actives, for example, Duchenne Muscular Dystrophy; (3) conditions that are progressive without curative treatment except symptom management that may extend over many years, for example, severe metabolic conditions; (4) conditions that are irreversible but non-progressive causing severe disability leading to health complications and premature death, for example, cerebral palsy [2]. These conditions may span over many years and include a gradual decline in health and function which can be distressing for families to witness and can affect the family’s daily life and routines [3–6]. Parents may need to shift family resources and time to meet the ill child’s needs, leaving less time or ability to meet the needs of the ill child’s siblings [7].

When a child is first diagnosed with a life-limiting condition, siblings may experience intense emotional and psychological stress [8]. It can be especially difficult during the first month after diagnosis; however, Sharpe et al. and Houtzager et al. reported these problems may subside six months after diagnosis [9, 10]. The difficulties that siblings experience early on is more evident among siblings of children with cancer and less so with siblings of children with other chronic conditions [11, 12]. The process of seeking and waiting for a diagnosis can be distressing and may take years, particularly for children eventually diagnosed with a progressive life-limiting condition (e.g., category 3 in the Together for Short Lives definition) [13]. When examining the sibling experience in this population, it may be more relevant to examine the sibling experience in relation to the timing of when the child became ill or when the parents first sought treatment for the child as opposed to when the child received a confirmed diagnosis. However, this has not yet been examined [14].

Previous research indicates healthy siblings of children with a life-limiting condition are at risk for poor overall emotional and behavioural well-being compared to siblings of healthy children [8, 9, 15, 16]. Some siblings’ psychosocial problems may be temporary, while others may last for years, impacting their ability to cope and navigate through life at home and in other social settings [14, 17, 18]. While recognizing that many factors may impact well-being, siblings’ sex and birth order in relation to the ill child have most commonly been examined for impact on the psychosocial well-being of siblings [19, 20]. For example, brothers have been found to mostly exhibit behavioural problems, whereas sisters reported more emotional problems in families of children with cancer. This is consistent with Sharpe et al. and Read et al., who found that sisters took on more caregiving responsibilities early on, which led to anxiety and depression [9, 10, 21, 22]. However, Barrera et al. found that parents reported more behavioural issues in sisters than brothers among children with cancer [23]. In terms of birth order, siblings who were older than the child diagnosed with cancer were found to have high levels of emotional distress, while siblings younger than the ill child had more behavioural problems in some studies [22, 24, 25]. However, Long, Alderfer et al. reported that siblings who were younger than the child with cancer had more distress than siblings who were older [26]. These inconsistencies suggest a need for more research on the impact of birth order and sex on psychosocial problems. Additionally, much of the existing research has focused on the experiences of siblings of children with cancer, which may not be representative of siblings of children with other types or trajectories of life-limiting conditions. This significantly limits our understanding of potential differences or unique aspects of siblings’ experiences based on differences in illness trajectory that might guide approaches to meeting support needs [27].

### Aim

The overall aim of this study was to examine psychosocial trajectories in siblings of children living with progressive life-limiting conditions and factors (the role of time since first seeking treatment for the ill child’s symptoms, sex, and birth order) that influence these trajectories over time.

### Methods

#### Design and population

We conducted a secondary analysis of a subset of sibling participants from the Charting the Territory (CTT) study [28]. Briefly, the CTT study aimed to document and determine the experiences of families of children with progressive, life-limiting metabolic, neurological, or chromosomal conditions [28]. Children and their families were recruited from nine clinical sites (seven in Canada, two in United States) between July 2009 and October 2012, and data collection occurred concurrently with recruitment. Designated parents completed baseline and follow-up questionnaires every 6 months. If there were two or more siblings in the same family, the designated parent completed questionaries separately for each sibling. All data was anonymized prior to this secondary analysis and no identifying information from participants could be extracted. The data was stored in an encrypted file and shared only with research team members who were actively involved in this study. Complete details of the CTT study can be found elsewhere [28].

The CTT study was approved by the University of British Columbia/Children’s and Women’s Health Center of British Columbia Research Ethics Board (certification no. H08-00124) and the participating study sites’ ethics boards. Informed consent was obtained from all participants by the CTT study team. The secondary analysis reported here received ethics approval from the University of Toronto Research Ethics Board (Protocol # 00037570).

#### Inclusion and exclusion criteria

Families were recruited through convenience sampling and were eligible to take part if:

(1) they had one or more children aged 19 years or less who had a progressive life-limiting condition, (2) biological, adoptive, or step-siblings aged 7 to 18 years who had no cognitive impairment and/or severe health conditions and lived with the ill child, (3) at least one biological, adoptive, step-parent, or legal guardian of the ill child needed to be willing to complete the surveys, and (4) they were able to speak and write English or French. Exclusion criteria were: (1) children who had potentially curative therapies, (2) children with CNS impairment due to hypoxic-ischemic injury, and (3) children in foster care. Families were not approached to participate or contacted for the follow-up questionnaires if the ill child was imminently dying.

### Measures

For the purposes of this secondary analysis, only demographic information collected from parents at study enrollment and siblings’ behaviours as reported by parents every six months were used. While data were collected up to seven times from each participating family, there was significant missing data at later time points. Some families were only recruited into the study a year before it ended, some families moved to a different city, or the ill child died at which point data collection was suspended for at least six months. Thus, only data collected from 58 eligible families at the first three time points (i.e., baseline – Time 1, six months – Time 2, 12 months – Time 3) in the larger study were used for this analysis. Missing data was handled with Full Information Maximum Likelihood (FIML) that was considered to be robust and sufficient.

#### Demographic questionnaire

Members of the original study team developed a demographic questionnaire which included items about the child’s diagnosis, siblings’ sex and age, total number of children in the family, birth order of siblings in relation to the ill child, parents’ education level, parents’ marital status, and average household income [28, 29]. Given previous research in other populations suggesting that time since diagnosis may be a factor that influences sibling’s experience, e.g., Yang et al., we were interested in exploring this factor in siblings of children with progressive life-limiting conditions [16]. However, significant symptoms may be identified in a child long before a diagnosis is made and in some cases a diagnosis may not be confirmed until after the child’s death, if ever. Thus, parents were asked to indicate the date when they first sought medical treatment after noticing something might be wrong with their ill child. We subtracted this date from the date of study enrolment to create a “Time Since First Sought Treatment” variable instead of time since diagnosis. Responses were categorized as (1) less than or equal to one year or (2) greater than one year. Sibling birth order (1 = older than ill child or 2 = younger than ill child) and sex (1 = male or 2 = female) were also examined in terms of their relationship with healthy siblings’ observed behaviour over time.

#### Siblings’ emotional and behavioural problems

Parents reported siblings’ behavioural and emotional problems using the Child Behaviour Checklist (CBCL) [30]. One parent per family completed the initial and follow-up CBCL for each participating sibling (e.g., if two siblings from the same family took part, the parent would complete the CBCL for each sibling separately). To operationalize healthy siblings’ behavioural and emotional problems over time, we used three composite scales, Internalizing, Externalizing, and Total Behaviour Problems [31]. The CBCL was scored according to the guidelines in Achenbach and Rescorla’s CBCL manual [30]. Each item in the CBCL is rated on a 3-point scale (0= “Not true”, 1= “Somewhat or sometimes true”, 2= “Very or often true”). Internalizing Problems Scale measured emotional problems within the child, and consisted of three subscales: *Anxiety/Depression*,* Withdrawal*,* and Somatic Complaints*. There were 32 items in these subscales with a total raw score ranging from 0 to 64. Externalizing Problems Scale focused on observable behaviours, and it measured a child’s conflicts with other people. It consists of two subscales: *Rule-Breaking Behaviour and Aggressive Behaviour.* These two subscales consist of 35 items with a total raw score ranging from 0 to 70. The Total Behaviour Problem score is calculated by adding Internalizing and Externalizing Problems scores plus scores from three additional subscales: *Thought*,* Attention*, and *Social and Other Problems*. The Total Behaviour Problems raw score ranges from 0 to 240. Reliability and validity of the CBCL composite scale in similar populations has been documented [10]. Stephenson, DeLongis previously reported Cronbach’s alpha for each scale in the current sample and demonstrated good internal consistency [32]. As recommended by Achenbach & Rescorla, we calculated T-scores for each scale to facilitate comparisons amongst the scale scores [30]. The developers also established cut off scores based on the distribution of CBCL scores when administered to healthy children. T-scores greater than 63 (90th percentile) are categorized as ‘clinical range’, while T-scores 60–63 (84th to 90th percentiles) are classified as ‘borderline range’, and T-scores less than 60 identified as ‘normal range’. A score in the clinical range suggests that sibling may require professional help; while a score in the borderline range suggests there are problems but not to the extent that siblings need professional help.

### Data analysis

Prior to calculating the means and standard deviations of the healthy siblings’ Internalizing, Externalizing, and Total Behaviour Problems at Time 1 (baseline), Time 2 (6 months), and Time 3 (12 months), we assessed our data distribution for normality. We computed percentages to report the proportion of healthy siblings with scores in the normal, borderline, and clinical ranges at each time point using SPSS Version 29 [33].

To investigate the effects of time and sex, birth order, and time since first sought treatment on the outcome variables, we performed three separate linear mixed effects analyses. In each analysis corresponding to an outcome variable – internalizing, externalizing, and total problems – we included sex, birth order, time since first sought treatment, and time point as fixed effects, individual ID and family ID as random effects. Internalizing, externalizing, total problems were treated as continuous variables. This was done to account for the repeated measures for each sibling, as well as the hierarchical family structure for siblings who belong to the same family. We further investigated multicollinearity between gender, birth order, and time since first sought treatment using variance inflation factor (VIF). VIF values around 1 suggest that there is no multicollinearity, thus allowing us to include all predictors in the same model.

## Results

A total of 258 families were recruited into the larger study with 370 siblings from 197 families who had more than one child. Of the 370 siblings, 61 siblings did not respond to the study invitation. Of the remaining 309 siblings’ only 121 siblings met eligibility criteria. There were 92 siblings who were under the ages of 7, 58 were above 18 years, 18 had cognitive impairment, and 15 had severe health conditions. A total of 56 siblings declined further contact. An additional five siblings were recruited during the study. There were 70 siblings from 58 families in the final sample. A detailed enrollment process can be found elsewhere [32].

Family and sibling demographic characteristics are summarized in Table 1. Of the 70 healthy siblings, 44.3% were male, 65.7% were older than the ill child, and 81% came from the 47 families who had an ill child living with a progressive life-limiting condition for longer than one year. The mean scores of Internalizing, Externalizing, and Total Behaviour Problems at each time point were found to be normally distributed. Therefore, means and standard deviations (SD) were reported in Table 2 and the results are also presented in a graph (Supplementary Material 1). The highest mean score (mean = 53.7, SD = 10.1) was reported for Internalizing Problems at Time 1, and the lowest mean score (mean = 48.2, SD = 10.0) was reported for Externalizing Problems at Time 3. The Internalizing, Externalizing, and Total Behaviour Problems scores were categorized into Normal, Borderline, or Clinical (see Table 2), where 25% of the healthy siblings had scores in the clinical range for Internalizing Problems at Time 1. For each behaviour problem, the proportion of siblings in the clinical range decreased over the three time points. The proportion of siblings’ problems are also presented as graphs in Supplementary Material 1.

**Table 1:** Sample characteristics at baseline

Characteristics	*n* (%)
Family Information, *N* = 58	
Average Household Income (Canadian Dollars)*	
< $40,000	13 (23.2)
$40,000 - <$80,000	20 (35.7)
$80,000 - $120,000	16 (28.6)
>$120,000	7 (12.5)
Total Number of Children Per Family**	
2	26 (44.8)
3	12 (20.6)
4	15 (25.8)
* ≥* 5	5 (8.6)
Number of Participating Siblings Per Family	
1	48 (82.8)
> 1	10 (17.2)
Child Information, *N* = 58	
Time Since First Sought Treatment***	
≤ 1 year	11 (19.0)
> 1 year	47 (81.0)
Child’s Diagnosis	
Severe neurological impairment – not yet diagnosed	13 (22.4)
Epileptic encephalopathy/neurodegenerative disease	10 (17.2)
Mitochondrial encephalomyopathy	10 (17.2)
Lysosomal/Peroxisomal leukodystrophy	8 (13.8)
Multi-organ congenital abnormalities	6 (10.3)
Others^	11 (19.0)
Sibling Information, *N* = 70	
Sibling Sex	
Male	31 (44.3)
Female	39 (55.7)
Sibling Age, Mean/Standard Deviation (SD)	11.2/3.2
Birth Order of Siblings^┼^	
Older than the ill child	46 (65.7)
Younger than the ill child	24 (34.3)

*percentages do not add up due to missing data

**The total number of children per family includes the child with a progressive metabolic, genetic, and neurological condition

***Time since first sought treatment refers to the duration between siblings’ study enrolment and when parents first sought medical treatment after noticing something was wrong with their ill child

^Others: Structural CNS abnormalities, Neuromuscular disease, small molecules disease, other conditions not specified

^┼^Birth order of siblings refers to the siblings’ rank in relation to the ill child i.e., older than or younger than the ill child

**Table 2:** CBCL scores and percentage of scores classified as normal, borderline, or clinical in healthy siblings (*N* = 70) at each time point

	Internalizing problems	Externalizing problems	Total behaviour problems
Time 1			
Mean* (Standard Deviation)	53.7 (10.1)	51 (10.7)	52.6 (10.8)
Percentage of CBCL Scores, n (%)			
Normal	50 (72)	59 (84)	54 (77)
Borderline	2 (3)	1 (2)	4 (5)
Clinical	18 (25)	10 (14)	12 (18)
Time 2			
Mean* (SD)	52.3 (9.6)	50.2 (10.7)	51 (9.7)
Percentage of CBCL scores, n (%)			
Normal	57 (81)	59 (84)	59 (84)
Borderline	1 (2)	3 (4)	3 (4)
Clinical	12 (17)	8 (12)	8 (12)
Time 3			
Mean* (SD)	50.4 (10.7)	48.2 (10.0)	48.8 (10.5)
Percentage of CBCL scores, n (%)			
Normal	63 (90)	64 (91)	59 (84)
Borderline	2 (3)	0 (0)	6 (9)
Clinical	5 (7)	6 (9)	5 (7)

*The CBCL mean scores reported in this table refer to the T-scores

*Note* The mean T-scores in the normative sample is 50 with a standard deviation of 10

### Factors related to internalizing problems among siblings

The maximum VIF for all covariates included in our model was 1.06 indicating that there was minimal multicollinearity. Results of all the models (i.e., Internalizing, Externalizing, and Total Behaviour Problems) are reported in Table 3. Linear mixed models showed that there was a significant effect of time *F* (2, 33) = 9.27, *p* = .001 and siblings’ sex *F* (1, 45) = 2.45, *p* = .012 on Internalizing Problems. The interaction between time and time since first sought treatment was statistically significant *F* (2, 33) = 5.10, *p* = .012.

**Table 3:** Mixed linear model analyses of siblings’ sex, birth order, and time since first sought treatment on internalizing, externalizing, and total behaviour problems

	Internalizing	*p*-values	Externalizing	*p*-values	Total Behaviour	*p*-values
Sex	2.44	0.012*	1.80	0.001*	0.43	0.516
Time since first sought treatment	0.83	0.367	0.45	0.02*	1.48	0.231
Birth order	0.01	0.922	0.19	0.826	1.06	0.013*
Time	9.27	0.001*	1.48	0.240	6.99	0.003*
Time*birth order	1.83	0.176	0.86	0.493	0.33	0.003*
Time*sex	1.79	0.454	1.02	0.370	4.17	0.023*
Time*Time since first sought treatment	5.10	0.012*	1.23	0.304	1.23	0.002*

*Note* The *f*-statistics of each variable and its corresponding *p*-values are reported in this table

*p-values < 0.05

### Factors related to externalizing problems among siblings

The model showed a significant effect of siblings’ sex *F* (1, 47) = 1.80, *p* = .001 and time since first sought treatment *F* (1, 44) = 0.45, *p* = .002 on Externalizing Problems. No interactions between time and siblings’ sex, birth order, and time since first sought treatment were identified.

### Factors related to total behaviour problems among siblings

For Total Behaviour Problems, we found a significant effect of time *F* (2, 34) = 6.99, *p* = .003, and birth order *F* (2, 17) = 1.06, *p* = .013 on Total Behaviour Problems which indicates a decrease in Total Behaviour Problems at 6 months and 12 months. We also found significant effects on the following three interaction terms: (1) time and siblings’ sex *F* (1, 47) = 4.17, *p* = .023, (2) time and time since first sought treatment *F* (4, 33) = 1.23, *p* = .002, and (3) time and siblings’ birth order *F* (2, 16) = 0.326, *p* = .003.

## Discussion

This study examined the relationship between siblings’ sex, birth order, and time since first sought treatment and internalizing, externalizing, and total behaviour problems over time. We found these problems improve with time and found significant relationships between sex, birth order and time since first sought treatment on the outcomes.

Siblings had behaviour problem scores that were in the clinical range, which was the largest at Time 1, and it decreased by Time 3. This proportion was greater than those reported in other research. For instance, Humphrey et al. examined Internalizing and Externalizing Problems in 30 siblings of children with a variety of life-limiting conditions (e.g., neuromuscular, genetic/congenital, and metabolic, conditions) using the Behavioural Assessment System for Children, Second Edition (BASC-2), a similar tool to the CBCL [34]. They found that 7% of siblings had Internalizing and Externalizing Problem scores in the clinical range. Similarly, Achenbach & Rescorla reported that 10% of the normative sample had Internalizing and Externalizing Problems scores within clinical range [30]. In contrast, we found the proportion of siblings at Time 1 with Internalizing Problems and Externalizing Problems were 2 to 3 times higher that reported by Humphrey et al. [34] However, at Time 3, the proportion of siblings within clinical range was similar to the normative sample and those in Humphrey et al.’s population [34].

The changes in siblings’ emotions and behaviour with time as observed in our study may be attributed to factors such as availability of support. Wawrzynski et al.’s review highlighted that having different supports such as informal family social support and informational support may be beneficial to siblings of children with cancer [35]. However, there was limited information on when supports should be provided directly to siblings. Furthermore, the trajectories of children diagnosed with cancer may be uniquely different compared to children diagnosed with progressive neurological, metabolic, and genetic conditions. To better care for siblings in the latter group, supports should be tailored to meet siblings’ specific needs, and also consider the timing of interventions in relation to the child’s illness.

Siblings of children with cancer and chronic illness generally exhibit more problems closer to the time of diagnosis [24, 36, 37]. In our study it was necessary to use a slightly different concept focusing on the time when parents first sought treatment for their ill child. However, findings were similar in that siblings had higher Internalizing and Externalizing Problems closer to the time that the child first became ill enough for parents to seek help compared with those who had been living with an ill child in the family for more than one year. These findings suggest that providing support to siblings early in the disease process may help them understand and better cope with upcoming challenges.

Siblings who were older than the ill child had higher Total Behaviour Problems compared to siblings who were younger than the ill child. A possible explanation may be that older siblings remember a time before the ill child was born or became ill and experience a significant change in their lives. Younger siblings may have only known their family as one that includes an ill child thus not be aware or have experienced a particular change in their life due to the illness. As well, parents spend significant time caring for their child with life-limiting conditions and assume older siblings are mature enough to cope on their own. However, Yang et al. found that older siblings still require appropriate support and guidance to understand their unique situation [16]. Our findings were contrary to Alderfer et al.’s review where they found younger siblings had higher levels of Internalizing and Externalizing Problems than older siblings [8]. However, their focus was on siblings of children with cancer who may have a different experience than siblings of children with progressive life-limiting conditions. Further research is needed to understand the role of siblings’ birth order in shaping their experience of having a brother or sister with progressive life-limiting conditions.

Two interesting findings that surfaced were: (1) brothers had higher Internalizing Problems than sisters, and (2) sisters had higher Externalizing Problems than brothers. Though the differences between brothers’ and sisters’ Internalizing and Externalizing Problems scores were small, Achenbach & Rescorla indicated that a change in scale scores is considered significant when group scores are statistically different [30]. While some authors found that brothers were more likely to develop Externalizing Problems, others found that brothers may experience emotional struggles as they tend to receive less illnes related communication from parents than sisters [10, 24, 38]. Also, researchers found that sisters generally were more prone to internalizing problems likely due to increased caregiving responsibilities at home, less social activities with peers, and lack of parental attention [37, 39]. However, our findings revealed that sisters had more externalizing problems compared to brothers possibly due to uncertainties in the initial phases of the child’s condition. This was supported in Malcolm et al.’s study that suggests sisters’ externalizing problems may be an effort to gain greater parental attention as parents tend to focus on the child with life-limiting conditions [40]. Similar to birth order, further research is needed to examine if there are differences in emotional and behavioural outcomes among healthy siblings based on both sex and gender. It may be particularly useful to conduct in-depth interviews to elicit siblings’ perceptions of the challenges they face.

## Limitations

Despite the strengths of using a longitudinal design, there are some noteworthy limitations. First, this study examined only the emotional and behavioural aspects of siblings’ experiences measured by CBCL. There may be other important aspects of siblings’ experiences, such as empathy, growth, or resilience, that are not captured by the tool. Second, families coping well may have been more likely to agree to participate in this research than families who may have been overwhelmed and thus reluctant to participate in a longitudinal study. This potential response bias would reduce the generalizability of our findings but may represent an underestimate of the degree of emotional and behavioural problems among healthy siblings. Third, while we accounted for three key variables - birth order, sex, and time since first sought treatment - other factors may also influence siblings’ emotional and behavioural problems, such as the severity of illness, types of support that siblings received, parents’ level of stress, or siblings’ perception of coping. While the sample size in our study was sufficient to complete our planned analysis, it was relatively small in terms of generalizability. Larger samples are required to be able to account for a wider variety of variables. Finally, this study examined siblings’ behaviour only from parents’ perspectives. While parents may provide accurate observation and information about their children’s problems, Houtzager et al. showed discrepancies between parental reports vs. sibling self-reports [41]. As such, future studies should examine siblings’ behaviour from multiple informants such as siblings themselves, parents, and teachers at school to determine if emotional and behavioural trajectories differ from those identified in this study. Having multiple informants may help to provide a clearer and more consistent picture of siblings’ behaviour trends in different settings such as school and home [41, 42]. Although data collected for this study was more than a decade ago, given the lack of longitudinal studies on this unique population, our information provides valuable insight into siblings’ emotions and behaviour through their parents’ perspectives.

### Implication for practice

Healthy siblings are known as the ‘forgotten mourners’ and often the provision of support for them may be delayed or limited [19]. Our study showed that siblings experience emotional and behavioural challenges early in the child’s illness trajectory. It may be helpful that healthcare providers work with parents and consider what supports might be useful for siblings. While most pediatric hospitals have specialized and trained staff such as child life specialists or play therapists, support programs are mainly designed for pediatric patients whose needs may be different compared to their healthy siblings. It is helpful to consider developing unique support programs for healthy siblings of children with progressive life-limiting conditions that use a holistic approach to address both emotional and behavioural challenges related to a high degree of uncertainty inherent in the disease course.

## Conclusion

While most siblings of children with a progressive life-limiting condition had Internalizing, Externalizing, and Total Behaviour Problem scores within the normal range, some had scores in the clinical range. These behaviour problems were especially observed in siblings in the first year after parents sought treatment for the ill child. Nevertheless, it is comforting to know that siblings’ behaviour problems declined with time and Internalizing Problems improved at a faster rate than Externalizing Problems. It is important to continue uncovering other social aspects such as family-, school-, and community-related factors that may play an important role in supporting siblings with their behaviour during the ill child’s illness trajectory as these factors may have implications for siblings’ well-being both at home and at school.

### Electronic supplementary material

Below is the link to the electronic supplementary material.

Supplementary Material 1

Supplementary Material 2

## Data Availability

No datasets were generated or analysed during the current study.
