# Supplementary material for: Examining emotional and behavioural trajectories in siblings of children with life-limiting conditions
Source: BMC Palliat Care. 2024 Aug 12;23:205. doi: 10.1186/s12904-024-01535-y (PMC11318302; doi:10.1186/s12904-024-01535-y)
Supplement: Supplementary file 1 — Supplementary Material 1 [file 12904_2024_1535_MOESM1_ESM.docx]

**Supplemental Material 1**

*The CBCL mean scores reported in this graph refer to the T-scores.

*Note.* The mean T-scores in the normative sample is 50 with a standard deviation of 10.
