# Supplementary material for: Examining emotional and behavioural trajectories in siblings of children with life-limiting conditions
Source: BMC Palliat Care. 2024 Aug 12;23:205. doi: 10.1186/s12904-024-01535-y (PMC11318302; doi:10.1186/s12904-024-01535-y)
Supplement: Supplementary file 2 — Supplementary Material 2 [file 12904_2024_1535_MOESM2_ESM.docx]

**Supplemental Material 2**

This graph shows the proportion of siblings with varying levels (normal, borderline, and clinical) of Internalizing, Externalizing, and Total Behaviour problems at Time 1.

This graph shows the proportion of siblings with varying levels (normal, borderline, and clinical) of Internalizing, Externalizing, and Total Behaviour problems at Time 2.

This graph shows the proportion of siblings with varying levels (normal, borderline, and clinical) of Internalizing, Externalizing, and Total Behaviour problems at Time 3.
